# Comparison of Adverse Events Following Immunosuppressant Administration for Pediatric Patients With Renal Transplants Categorized by Two-Year Age Increments Using the U.S. Food and Drug Administration Adverse Event Reporting System

**DOI:** 10.7759/cureus.65300

**Published:** 2024-07-24

**Authors:** Toru Ogura, Chihiro Shiraishi, Yuko Tamura, Aiko Urawa

**Affiliations:** 1 Clinical Research Support Center, Mie University Hospital, Tsu, JPN; 2 Department of Pharmacy, Mie University Hospital, Tsu, JPN; 3 Faculty of Medicine, Mie University School of Nursing, Mie University, Tsu, JPN; 4 Organ Transplantation Centre, Mie University Hospital, Tsu, JPN

**Keywords:** database, large sample, linear regression analysis, mycophenolate mofetil, prednisone, tacrolimus

## Abstract

Background

Immunosuppressants are frequently administered to prevent transplant rejection in patients with renal transplants but cause various adverse events. The incidence of each adverse event may differ between pediatric and adult patients with renal transplants. Because the development of organs and bodies in pediatric patients varies greatly annually, the incidence of each adverse event following immunosuppressant administration may vary by age. Consequently, the age-specific incidence of each adverse event in pediatric patients represents invaluable information for clinical settings. To clarify trends in the occurrence of adverse events by age, a large sample size for each age is required. However, it is difficult to conduct clinical trials in pediatric patients with renal transplants with a large sample size for each age. One method to address this difficulty is to use a database.

Objectives

This study aimed to investigate the trends in the occurrence of each adverse event following immunosuppressant administration in pediatric patients with renal transplants, categorized by two-year age increments.

Methods

We extracted data on pediatric patients aged 0-17 years who received immunosuppressants after renal transplant between January 2004 and March 2024 from the U.S. Food and Drug Administration Adverse Event Reporting System. Because adverse events were greatly affected by age, the patients were divided into groups by two-year age increments. We analyzed the relationship between the groups and the reporting proportion of each adverse event by using the reporting regression coefficient (RRC) from univariate regression analysis and the adjusted RRC (aRRC), which controlled for differences in patient background.

Results

Renal tubular necrosis, renal impairment, chronic allograft nephropathy, and headache were the adverse events that required more attention with increasing age because RRC and aRRC were significantly > 0. By contrast, Epstein-Barr virus infection was the adverse event that required attention, especially in younger pediatric patients, because RRC and aRRC were significantly < 0. Additionally, there were various trends among other adverse events, including those that required careful monitoring across all ages 0-17 years.

Conclusions

This study demonstrated that the types of adverse events requiring attention in pediatric patients with renal transplants differ by age. These findings can help enhance treatment and care in pediatric clinical settings.

## Introduction

Immunosuppressants prevent transplant rejection and are often administered immediately after a renal transplant. Nine primary types of immunosuppressants are available: prednisone (including prednisolone and methylprednisolone) [1,2], tacrolimus [1,2], mycophenolate mofetil [1,2], cyclosporine [1,2], everolimus [1,2], azathioprine [1], rituximab [3], mizoribine [4], and basiliximab [1,2]. The types of adverse events occurring in pediatric patients with renal transplants may differ depending on their age. Previous studies have divided pediatric patients into age groups of 0-1, 2-5, 6-12, and 13-17 years [5]; however, the development of organs and bodies in pediatric patients varies greatly yearly [6]. Therefore, categorizing pediatric patients aged 6 and 12 years in the same group may be unreasonable. Studies outside the transplant field have demonstrated the value of dividing pediatric patients into finer age groups for large samples [7].

To study a large sample, we obtained data from the U.S. Food and Drug Administration Adverse Event Reporting System (FAERS) [8] that included adverse events reported worldwide since the first quarter of 2004 (2004Q1). FAERS enabled the analysis of the trends in the occurrence of each adverse event in pediatric patients at two-year age increments. FAERS data include variables such as patient background, administered drug names, reason for administration, and adverse event type. These variables allowed the selection of pediatric patients with renal transplants and the investigation of each adverse event following immunosuppressant administration. FAERS data have been used to investigate the occurrence of each adverse event following immunosuppressant administration to patients with various organ transplants [9,10].

Known adverse events following immunosuppressant administration include post-transplant lymphoproliferative disorder, renal tubular necrosis, renal impairment, toxic nephropathy, chronic allograft nephropathy, proteinuria, Epstein-Barr virus infection, BK virus infection, polyomavirus-associated nephropathy, polyomavirus viremia, cytomegalovirus infection, urinary tract infection, anemia, hypertension, hypotension, leukopenia, neutropenia, increased blood creatinine, headache, abdominal pain, diarrhea, vomiting, tachycardia, pyrexia, fatigue, mouth ulceration, hyperglycemia, and diabetes mellitus [11-18]. Because adverse events in FAERS are sometimes provided in British English, we used the spelling of adverse event types found in the FAERS database to avoid confusion.

## Materials and methods

Data source

The FAERS database has been unlinkable, anonymized, and made publicly available quarterly since January 2004. The database was started as the Adverse Event Reporting System (AERS) in the first quarter of 2004 (2004Q1) and continued until 2012Q3. In 2012Q4, it transitioned to the FAERS database, which includes a broader range of data than AERS. The AERS and FAERS data files, named aers_ascii_yyyyQq.zip and faers_ascii_yyyyQq.zip, respectively, were downloaded on May 4, 2024, where yyyy and q represent the year and quarter, respectively. The differences between AERS and FAERS data were reconciled based on their respective descriptions. Hence, all subsequent mentions of FAERS data include AERS data. The FAERS database consists of seven files, five of which were used in this study: patient demographic and administrative information (DEMOyyQq.txt, where yy represents the last two digits of the year), drug information (DRUGyyQq.txt), adverse event information (REACyyQq.txt), drug therapy start and end dates (THERyyQq.txt), and indications for use (INDIyyQq.txt). When new information is added to the existing data in FAERS, the existing data in the database is updated by incrementing the safety report version number {caseversion} rather than by overwriting. Therefore, only the highest {caseversion} number was used. Throughout this study, the names of the data elements used in FAERS are indicated using the curly braces convention. In the AERS data, {caseversion} was not provided; however, this judgment could be made using the unique number for identifying {ISR} and the case identification number {CASE}. Before analyzing {sex}, patient's age at the adverse event {age}, {weight}, and country of the reporter {reporter_country}, data-handling was performed, including responding to unexpected input and adjusting the units for {age} and {weight}. Data-handling techniques are presented in Appendix A. Additionally, because the AERS database did not always have line breaks inserted where required, line breaks were inserted at lines 322,967, 247,896, and 446,738 of DRUG11Q2.txt, DRUG11Q3.txt, and DRUG11Q4.txt before performing statistical analyses, respectively.

Approval from an institutional review board was not required because the FAERS is an unlinkable, anonymized database that is open to the public.

Study design

This study was classified as a cross-sectional investigation using the FAERS database.

The inclusion criteria were pediatric patients aged 0-17 years who received ≥ 1 immunosuppressant of interest after renal transplant between 2004Q1 and 2024Q1. Between 2014Q3 and 2024Q1, immunosuppressants in the FAERS database were identified by trade name using the variable for the product’s active ingredient, {prod_ai}. Between 2004Q1 and 2014Q2, where {prod_ai} was not provided, immunosuppressants were identified by trade and brand names shown in Appendix B by using the variable for the medical product, {drugname}. Of the database records extracted for the specified immunosuppressants, those records in which the data element describing the indication for use, {indi_pt}, was renal transplant were retained.

The exclusion criterion was that a patient had not been administered immunosuppressants before the occurrence of adverse events. If a patient had been administered immunosuppressants both before and after the occurrence of adverse events, we excluded only the data for immunosuppressants administered after the occurrence of the adverse events. Exclusion or retention was determined based on the date that the specific immunosuppressant was started (or re-started), {start_dt}, the date when the adverse event occurred or began, {event_dt}, and the date when the specific immunosuppressant was stopped, {end_dt}. Due to missing data for these three dates in many patients, we excluded only those patients for whom we could determine with certainty that immunosuppressants were started after the occurrence of adverse events. Details of this judgment are provided in Appendix C.

The endpoint was the occurrence of adverse events. This was provided as the preferred term {pt} level of medical terminology describing the event, using the Medical Dictionary for Regulatory Activities (MedDRA). The FAERS data, which is released quarterly, utilizes the most current version of MedDRA available at the time. The MedDRA version was not provided for the AERS data between 2004Q1 and 2012Q3. However, the MedDRA versions for the FAERS data between 2012Q4 and 2024Q1 were provided as follows: 2012Q4, version 16.0; 2021Q1-2013Q2, version 16.1; 2013Q3-2014Q2, version 17.0; 2014Q3, version 17.1; 2014Q4-2015Q2, version 18.0; 2015Q3-2015Q4, version 18.1; 2016Q1-2016Q2, version 19.0; 2016Q3-2017Q1, version 19.1; 2017Q2, version 20.0; 2017Q3-2017Q4, version 20.1; 2018Q1-2018Q3, version 21.0; 2018Q4-2019Q1, version 21.1; 2019Q2-2019Q3, version 22.0; 2019Q4-2020Q1, version 22.1; 2020Q2, version 23.0; 2020Q3-2021Q1, version 23.1; 2021Q2-2021Q3, version 24.0; 2021Q4-2022Q1, version 24.1; 2022Q2-2022Q3, version 25.0; 2022Q4-2023Q1, version 25.1; 2023Q2-2023Q3, version 26.0; and 2023Q4-2024Q1, version 26.1. The FAERS collects reports of adverse events that occur following drug administration, often considered drug-related adverse events. However, because the FAERS data are reports of adverse events and not side effects, they also include adverse events that are not drug-related. Transplant rejection was considered a transplant-related adverse event occurring because immunosuppressant administration could not prevent the event and not a drug-related adverse event. Because this study focused on pediatric patients with renal transplants, both transplant rejection and kidney transplant rejection in the FAERS data were treated as transplant rejection.

Nine groups were established based on two-year age increments: 0-1, 2-3, 4-5, 6-7, 8-9, 10-11, 12-13, 14-15, and 16-17 years.

Statistical analyses

Continuous and categorical data were summarized as medians with first and third quartiles and as frequency and reporting proportion (RP) [19], respectively. The RP was calculated as (number of patients in the category of interest) / (number of patients in the target group) × 100. Furthermore, univariate and multivariate linear regression analyses were performed, with the dependent variable being the RP of each adverse event and the independent variables being group, {sex}, United States, Germany, France, prednisone, tacrolimus, and mycophenolate mofetil. We used these three immunosuppressants and three {reporter_countries} as independent variables because they were the first to third most reported in each category. As the correlation between {age} and {weight} was high in pediatric patients with renal transplants and several missing data points were noted for {weight}, it was not used as an independent variable. To perform linear regression analysis, each variable was quantified as groups (1, 0-1 year; 2, 2-3 years; 3, 4-5 years; 4, 6-7 years; 5, 8-9 years; 6, 10-11 years; 7, 12-13 years; 8, 14-15 years; and 9, 16-17 years), {sex} (1, male; 0, female), United States (1, United States; 0, other countries), Germany (1, Germany; 0, other countries), France (1, France; 0, other countries), and each immunosuppressant (1, administration; 0, non-administration). We calculated the reporting regression coefficient (RRC) and its 95% confidence interval (95% CI) and adjusted the RRC (aRRC) and its 95% CI by using univariate and multivariate linear regression analyses, respectively. In aRRC, the group variable was always included, and the forward selection method was used to determine whether other variables were included. Variables with p < 0.05 were included in the model, whereas those with p ≥ 0.05 were not included. Statistical significance was considered at p < 0.05. The software R version 4.2.2 (R Foundation for Statistical Computing, Vienna, Austria) was used for statistical analyses.

Due to the absence of records reporting zero adverse events in the FAERS database, calculating the incidence of each adverse event was impossible. Therefore, similar to previous studies [9,10], this study also used the RP and RRC with the addition of “reporting” to differentiate the statistical analysis methods using FAERS data from the usual statistical analysis methods.

## Results

Patient background

**Figure 1:**
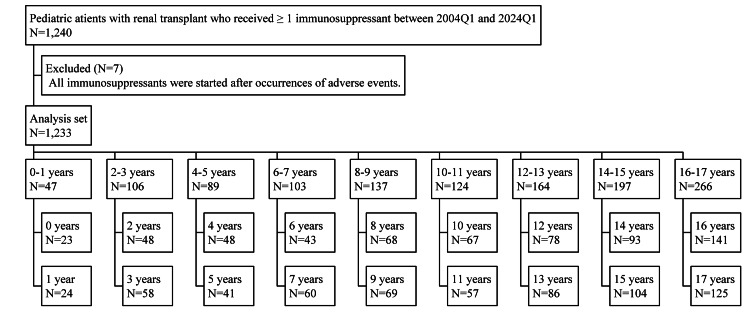
Flowchart of pediatric patients with renal transplants who received ≥ 1 immunosuppressant.

Between 2004Q1 and 2024Q1, we identified 1,240 pediatric patients with renal transplants who received ≥ 1 immunosuppressant. The analysis set consisted of 1,233 patients after excluding seven patients. Figure 1 shows the breakdown of each group from the analysis set. The sample sizes for two-year age increments were sufficient for statistical analyses. However, one-year age increments were considered desirable for analysis, but this was not performed due to the small sample for each one-year age increment. Table 1 summarizes the patient backgrounds of the nine groups. 

**Table 1:** Summary of patient background. Sex and country are summarized as frequency (reporting proportion). Age and weight are summarized as medians with first and third quartiles. Unknown for each variable is summarized as frequency (reporting proportion). Q1: first quartile; Q3: third quartile; RP: reporting proportion

	0-1 years	2-3 years	4-5 years	6-7 years	8-9 years	10-11 years	12-13 years	14-15 years	16-17 years
	N=47	N=106	N=89	N=103	N=137	N=124	N=164	N=197	N=266
Sex									
Female, n (RP)	12 (25.5)	45 (42.5)	37 (41.6)	39 (37.9)	60 (43.8)	37 (29.8)	73 (44.5)	74 (37.6)	104 (39.1)
Male, n (RP)	29 (61.7)	51 (48.1)	49 (55.1)	61 (59.2)	65 (47.4)	79 (63.7)	89 (54.3)	98 (49.7)	143 (53.8)
Unknown, n (RP)	6 (12.8)	10 (9.4)	3 (3.4)	3 (2.9)	12 (8.8)	8 (6.5)	2 (1.2)	25 (12.7)	19 (7.1)
Weight, kg									
Median	5.1	14.0	17.0	20.0	25.0	30.5	38.5	42.0	54.0
Q1-Q3	1.7-10.4	12.2-20.0	15.7-20.0	19.0-22.0	22.0-30.0	26.2-39.4	34.8-47.0	36.2-51.8	44.8-58.0
Unknown, n (RP)	31 (66.0)	61 (57.5)	60 (67.4)	72 (69.9)	107 (78.1)	94 (75.8)	114 (69.5)	143 (72.6)	218 (82.0)
Country									
United States, n (RP)	8 (17.0)	22 (20.8)	28 (31.5)	21 (20.4)	24 (17.5)	25 (20.2)	26 (15.9)	43 (21.8)	61 (22.9)
Canada, n (RP)	0 (0.0)	4 (3.8)	1 (1.1)	6 (5.8)	11 (8.0)	2 (1.6)	16 (9.8)	16 (8.1)	25 (9.4)
Colombia, n (RP)	1 (2.1)	4 (3.8)	2 (2.2)	3 (2.9)	7 (5.1)	6 (4.8)	5 (3.0)	17 (8.6)	17 (6.4)
Germany, n (RP)	9 (19.1)	15 (14.2)	12 (13.5)	9 (8.7)	21 (15.3)	8 (6.5)	14 (8.5)	42 (21.3)	25 (9.4)
Spain, n (RP)	0 (0.0)	1 (0.9)	3 (3.4)	4 (3.9)	2 (1.5)	1 (0.8)	12 (7.3)	2 (1.0)	8 (3.0)
France, n (RP)	13 (27.7)	15 (14.2)	15 (16.9)	10 (9.7)	6 (4.4)	8 (6.5)	19 (11.6)	7 (3.6)	20 (7.5)
United Kingdom, n (RP)	4 (8.5)	6 (5.7)	1 (1.1)	14 (13.6)	15 (10.9)	18 (14.5)	7 (4.3)	9 (4.6)	20 (7.5)
Sweden, n (RP)	6 (12.8)	6 (5.7)	3 (3.4)	2 (1.9)	2 (1.5)	0 (0.0)	1 (0.6)	4 (2.0)	1 (0.4)
Turkey, n (RP)	0 (0.0)	3 (2.8)	2 (2.2)	1 (1.0)	1 (0.7)	4 (3.2)	7 (4.3)	5 (2.5)	12 (4.5)
Japan, n (RP)	1 (2.1)	11 (10.4)	8 (9.0)	3 (2.9)	13 (9.5)	12 (9.7)	10 (6.1)	6 (3.0)	9 (3.4)
Others, n (RP)	3 (6.4)	8 (7.5)	8 (9.0)	23 (22.3)	15 (10.9)	30 (24.2)	25 (15.2)	23 (11.7)	44 (16.5)
Unknown, n (RP)	2 (4.3)	11 (10.4)	6 (6.7)	7 (6.8)	20 (14.6)	10 (8.1)	22 (13.4)	23 (11.7)	24 (9.0)
Immunosuppressant									
Prednisone, n (RP)	20 (42.6)	65 (61.3)	38 (42.7)	48 (46.6)	55 (40.1)	58 (46.8)	58 (35.4)	102 (51.8)	136 (51.1)
Tacrolimus, n (RP)	34 (72.3)	72 (67.9)	60 (67.4)	76 (73.8)	97 (70.8)	83 (66.9)	116 (70.7)	138 (70.1)	189 (71.1)
Mycophenolate, n (RP)	16 (34.0)	58 (54.7)	45 (50.6)	54 (52.4)	75 (54.7)	62 (50.0)	87 (53.0)	97 (49.2)	134 (50.4)
Cyclosporine, n (RP)	7 (14.9)	24 (22.6)	19 (21.3)	14 (13.6)	31 (22.6)	26 (21.0)	22 (13.4)	32 (16.2)	48 (18.0)
Everolimus, n (RP)	3 (6.4)	4 (3.8)	8 (9.0)	4 (3.9)	5 (3.6)	3 (2.4)	11 (6.7)	25 (12.7)	16 (6.0)
Azathioprine, n (RP)	9 (19.1)	7 (6.6)	5 (5.6)	7 (6.8)	1 (0.7)	10 (8.1)	3 (1.8)	10 (5.1)	19 (7.1)
Rituximab, n (RP)	1 (2.1)	1 (0.9)	1 (1.1)	2 (1.9)	10 (7.3)	2 (1.6)	8 (4.9)	2 (1.0)	2 (0.8)
Mizoribine, n (RP)	0 (0.0)	1 (0.9)	0 (0.0)	0 (0.0)	1 (0.7)	1 (0.8)	1 (0.6)	1 (0.5)	0 (0.0)
Basiliximab, n (RP)	4 (8.5)	16 (15.1)	11 (12.4)	15 (14.6)	20 (14.6)	11 (8.9)	10 (6.1)	14 (7.1)	54 (20.3)

Adverse events

**Figure 2:**
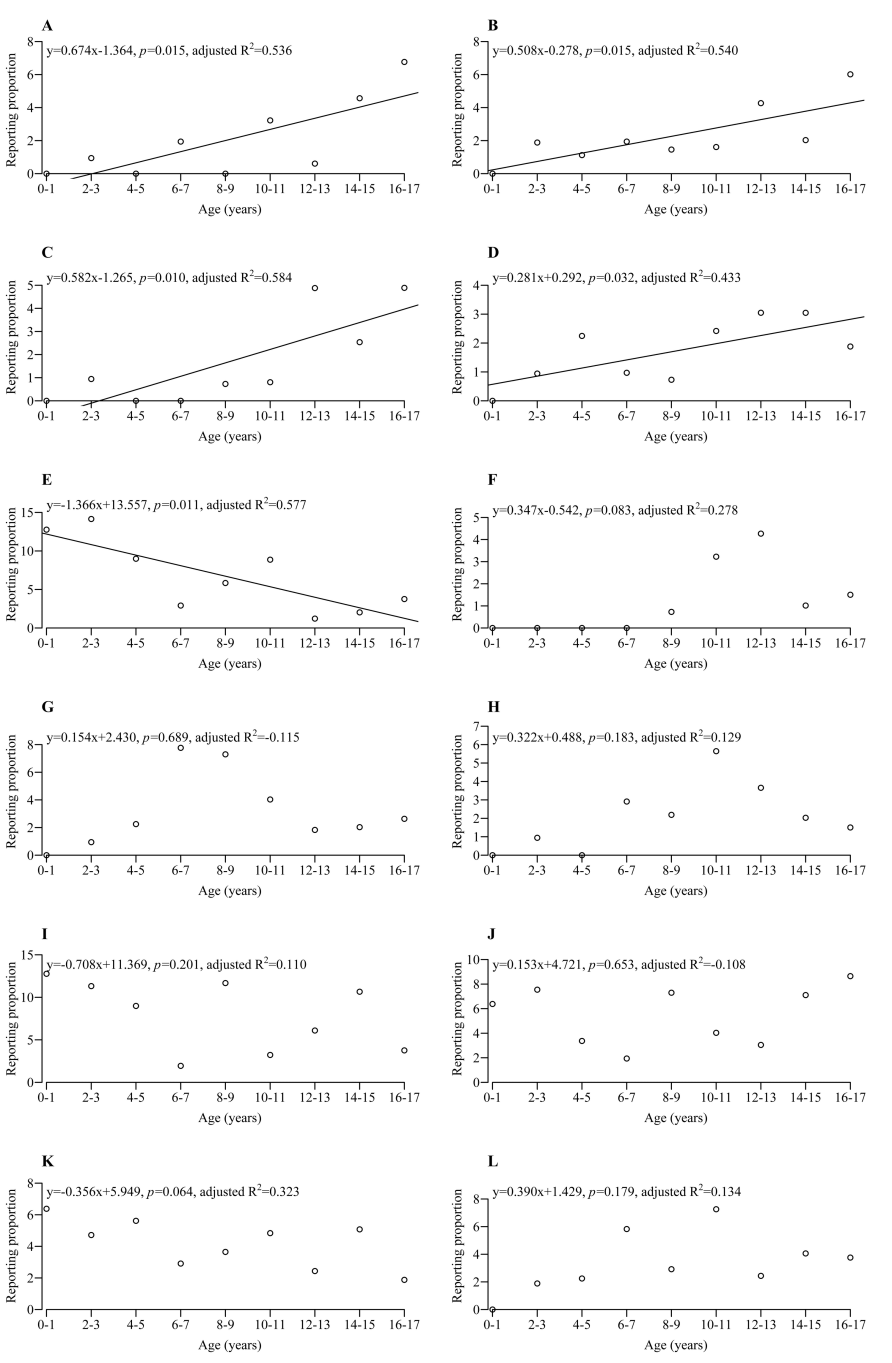
Scatterplot and regression line for each adverse event. (A) renal tubular necrosis; (B) renal impairment; (C) chronic allograft nephropathy; (D) headache; (E) Epstein-Barr virus infection, (F) mouth ulceration, (G) abdominal pain, (H) proteinuria, (I) BK virus infection, (J) polyomavirus-associated nephropathy, (K) polyomavirus viremia, and (L) cytomegalovirus infection Regression line was added if the RRC had p < 0.05 but not added if the RRC had p ≥ 0.05. R^2^: coefficient of determination

**Figure 3:**
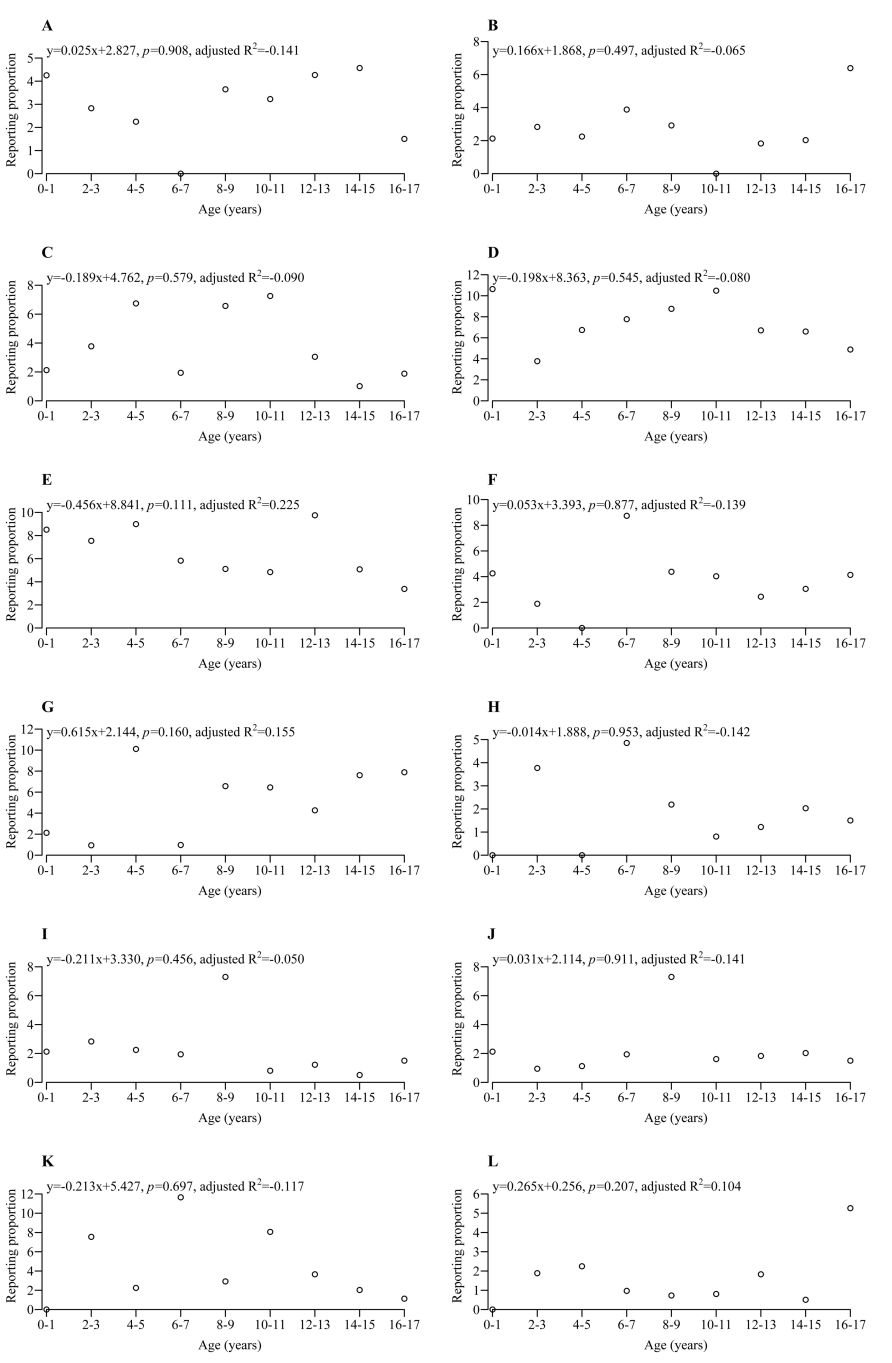
Scatterplot and regression line for each adverse event (continued from Figure 2). (A) urinary tract infection; (B) nephropathy toxic; (C) post-transplant lymphoproliferative disorder; (D) pyrexia; (E) diarrhea; (F) vomiting; (G) increased blood creatinine; (H) neutropenia; (I) leukopenia; (J) anemia; (K) hypertension; and (L) hypotension. Regression line was added if the RRC had p < 0.05 but not added if the RRC had p ≥ 0.05. R^2^: coefficient of determination

**Figure 4:**
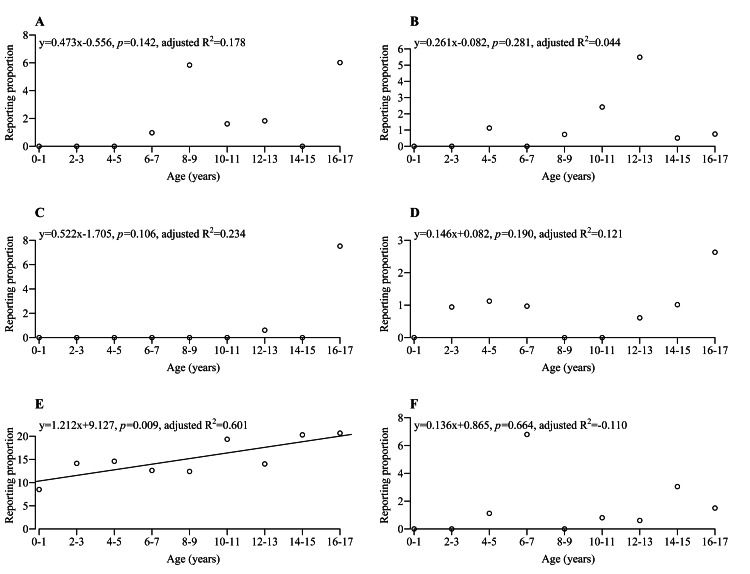
Scatterplot and regression line for each adverse event (continued from Figure 3). (A) Tachycardia, (B) fatigue, (C) hyperglycemia, (D) diabetes mellitus, (E) transplant rejection, and (F) treatment noncompliance. Regression line was added if the RRC had p < 0.05 but not added if the RRC had p ≥ 0.05. R^2^: coefficient of determination

Figures 2-4 show the scatterplot for the RP and the group in each adverse event. Further regression analysis was performed, and the regression line was added to the scatterplot if the RRC for the group was statistically significant. Table 2 shows detailed information on univariate and multivariate linear regression analyses for adverse events with statistical significance for both RRC and aRRC. The adverse events were categorized into five patterns based on their trends.

**Table 2:** Univariate and multivariate linear regression analyses. Each variable is quantified as follows: group (1, 0-1 year; 2, 2-3 years; 3, 4-5 years; 4, 6-7 years; 5, 8-9 years; 6, 10-11 years; 7, 12-13 years; 8, 14-15 years; and 9, 16-17 years), sex (1: male, 0: female), United States (1: United States, 0: other countries), Germany (1: Germany, 0: other countries), France (1: France, 0: other countries), and each immunosuppressant (1: administration, 0: nonadministration). -: not selected in multivariate linear regression analysis. aRRC: adjusted reporting regression coefficient; RRC: reporting regression coefficient

	Univariate		Multivariate	
Variable	RRC (95%CI)	p-value	aRRC (95%CI)	p-value
Renal tubular necrosis				
Group	0.674 (0.176 to 1.172)	0.015	0.757 (0.477 to 1.037)	0.001
Sex	0.003 (-0.363 to 0.370)	0.983	0.140 (0.002 to 0.277)	0.047
United States	0.069 (-0.392 to 0.531)	0.732	-	-
Germany	-0.079 (-0.493 to 0.334)	0.664	-	-
France	-0.170 (-0.410 to 0.070)	0.138	-	-
Prednisone	0.138 (-0.115 to 0.391)	0.237	0.184 (0.080 to 0.287)	0.006
Tacrolimus	0.029 (-0.901 to 0.959)	0.944	-	-
Mycophenolate	0.037 (-0.302 to 0.376)	0.802	-	-
Renal impairment				
Group	0.508 (0.135 to 0.880)	0.015	0.508 (0.135 to 0.880)	0.015
Sex	-0.068 (-0.337 to 0.201)	0.569	-	-
United States	-0.015 (-0.364 to 0.334)	0.922	-	-
Germany	-0.171 (-0.447 to 0.105)	0.186	-	-
France	-0.103 (-0.295 to 0.089)	0.244	-	-
Prednisone	0.014 (-0.197 to 0.225)	0.881	-	-
Tacrolimus	0.094 (-0.600 to 0.787)	0.759	-	-
Mycophenolate	0.117 (-0.117 to 0.350)	0.277	-	-
Chronic allograft nephropathy				
Group	0.582 (0.188 to 0.975)	0.010	0.582 (0.188 to 0.975)	0.010
Sex	-0.091 (-0.386 to 0.204)	0.491	-	-
United States	-0.090 (-0.470 to 0.290)	0.593	-	-
Germany	-0.096 (-0.436 to 0.243)	0.524	-	-
France	-0.095 (-0.316 to 0.126)	0.342	-	-
Prednisone	-0.018 (-0.252 to 0.216)	0.858	-	-
Tacrolimus	0.078 (-0.695 to 0.850)	0.819	-	-
Mycophenolate	0.076 (-0.200 to 0.352)	0.534	-	-
Headache				
Group	0.281 (0.032 to 0.531)	0.032	0.281 (0.032 to 0.531)	0.032
Sex	-0.014 (-0.180 to 0.151)	0.843	-	-
United States	0.061 (-0.142 to 0.265)	0.498	-	-
Germany	-0.050 (-0.235 to 0.135)	0.543	-	-
France	-0.073 (-0.184 to 0.038)	0.162	-	-
Prednisone	-0.016 (-0.143 to 0.111)	0.775	-	-
Tacrolimus	-0.196 (-0.579 to 0.187)	0.265	-	-
Mycophenolate	0.065 (-0.077 to 0.208)	0.315	-	-
Epstein–Barr virus infection				
Group	-1.366 (-2.302 to -0.430)	0.011	-1.366 (-2.302 to -0.430)	0.011
Sex	0.114 (-0.601 to 0.829)	0.717	-	-
United States	0.119 (-0.791 to 1.029)	0.766	-	-
Germany	0.207 (-0.598 to 1.013)	0.562	-	-
France	0.396 (-0.036 to 0.829)	0.067	-	-
Prednisone	0.229 (-0.285 to 0.744)	0.327	-	-
Tacrolimus	-0.855 (-2.519 to 0.809)	0.264	-	-
Mycophenolate	-0.278 (-0.902 to 0.345)	0.326	-	-

Pattern 1 exhibited an increasing trend with age. Both RRC and aRRC in the group were significantly > 0 (Table 2) for renal tubular necrosis (Figure 2A), renal impairment (Figure 2B), chronic allograft nephropathy (Figure 2C), and headache (Figure 2D). The RPs of these adverse events were low in younger pediatric patients but tended to increase significantly with age. Pattern 2 exhibited a decreasing trend with age. Conversely, both RRC and aRRC in the group were significantly < 0 (Table 2) for Epstein-Barr virus infection (Figure 2E). The RP of this adverse event was high in younger pediatric patients but tended to decrease significantly with age. In both univariate and multivariate linear regression analyses of these adverse events, variables other than the group rarely showed statistical significance (Table 2). Consequently, the RPs of these adverse events were found to be predominantly dependent on age. This suggests that age is the primary predictor of these adverse events, while other variables had minimal impact on the outcome.

Although the RRC had no statistical significance, some adverse events showed characteristic scatterplots. Pattern 3 exhibited a change in trend at a certain age. Mouth ulceration (Figure 2F) was not reported in patients aged 0-7 years but was occasionally reported in patients aged 8-17 years. Similar occurrence trends were observed for tachycardia (Figure 4A), fatigue (Figure 4B), and hyperglycemia (Figure 4C). These adverse events warrant increased vigilance in patients above a certain age. 

Pattern 4 exhibited a scatterplot with a mountain-like shape. Abdominal pain (Figure 2G) was higher in patients aged 6-7 and 8-9 years and lower in other ages. Proteinuria (Figure 2H) was higher in patients aged 10-11 years, moderate in patients aged 6-7, 8-9, 12-13 and 14-15 years, and lower in other ages. The scatterplot for these two adverse events showed a mountain-like shape.

Pattern 5 exhibited a flat trend regardless of age. Some adverse events were occasionally reported across ages 0-17, including BK virus infection (Figure 2I), polyomavirus-associated nephropathy (Figure 2J), polyomavirus viremia (Figure 2K), cytomegalovirus infection (Figure 2L), urinary tract infection (Figure 3A), nephropathy toxic (Figure 3B), post-transplant lymphoproliferative disorder (Figure 3C), pyrexia (Figure 3D), diarrhea (Figure 3E), vomiting (Figure 3F), increased blood creatinine (Figure 3G), neutropenia (Figure 3H), leukopenia (Figure 3I), anemia (Figure 3J), hypertension (Figure 3K), hypotension (Figure 3L), and diabetes mellitus (Figure 4D). These adverse events may need careful monitoring in pediatric patients with renal transplants, regardless of age.

Additionally, the scatterplot was drawn for transplant rejection (Figure 4E), which occurred despite immunosuppressant administration. The RRC of the group was significantly > 0 for transplant rejection. Furthermore, the scatterplot was drawn for treatment noncompliance (Figure 4F), which may be one of the factors leading to transplant rejection.

## Discussion

Post-renal transplantation care in pediatric patients necessitates vigilance for surgical complications [20], immune-mediated transplant rejection [21], and adverse events from immunosuppressant therapy [11-13]. Additionally, the continuous growth and development of organs and bodies in pediatric patients annually suggests that the incidence of adverse events following immunosuppressant administration may vary with age. Consequently, this study aimed to elucidate the trends of adverse events by categorizing pediatric patients into two-year age brackets, which yielded novel insights.

Renal tubular necrosis (Figure 2A), renal impairment (Figure 2B), chronic allograft nephropathy (Figure 2C), and headache (Figure 2D) were the adverse events with RP close to 0 at a young age but increased with age. This may be due to long-term immunosuppressant administration or related to the development of the body and organs [22,23]. However, we were unable to clarify the causal relationships because the FAERS data provides limited data and contains a lot of missing data. The reporting of subjective adverse events such as headaches and abdominal pain in pediatric patients aged 0-3 years may be challenging. Therefore, the number of reported subjective adverse events might be lower than the actual number. Conversely, the RP of Epstein-Barr virus infection (Figure 2E) [16] was higher at a younger age and decreased with age. Therefore, routine testing for Epstein-Barr virus infection is recommended for pediatric transplant patients aged 0-3 years. Some showed characteristic scatterplots in addition to the results where the graphs were upward- or downward-sloping. Some adverse events showed a mountain-like shape in the scatterplots, such as abdominal pain (Figure 2G) and proteinuria (Figure 2H), and some other adverse events showed a flat trend in the scatterplots, such as BK virus infection (Figure 2I) and polyomavirus-associated nephropathy (Figure 2J). Clarifying adverse events requiring attention by age might greatly contribute to clinical settings.

This study also assessed efficacy based on reports of transplant rejection. The RP of transplant rejection (Figure 4E) significantly increased with age. One reason is treatment noncompliance. The adverse events in the FAERS database included treatment noncompliance (Figure 4F). Treatment noncompliance was not reported in children aged 0-3 years, which may be due to parental management. Conversely, treatment noncompliance was occasionally reported in children aged 4-17 years, which may be due to increased self-management as they grow older. There were 20 reports of treatment noncompliance in patients aged 4-17 years, including 17 female patients, and 17 patients (2 males and 15 females) reported both treatment noncompliance and transplant rejection. Therefore, treatment noncompliance may have led to transplant rejection. Nevertheless, due to the limited sample size of patients exhibiting treatment noncompliance (n = 20), further research is warranted to elucidate the potential correlation between treatment noncompliance and transplant rejection. Female patients reporting more treatment noncompliance may be due to the desire of adolescent female patients to avoid adverse events from immunosuppressants. Previous studies have noted that puberty is a major risk factor for treatment nonadherence [24]. To avoid treatment noncompliance, these previous studies have recommended the simplification of medication regimens, providing in-depth education, promoting shared decisions among adolescents and parents, and involving other family members and friends. Another possible reason may be that younger pediatric patients (especially those aged 0-1 year) have less transplant rejection due to their immune system still developing [25], and the likelihood of transplant rejection may increase as they get older and their immune system develops.

The database for pediatric patients with renal transplants includes the North American Pediatric Renal Trials and Collaborative Studies and the Cooperative European Paediatric Renal Transplant Initiative registry [5,26,27]. Various studies were conducted using these databases, but they divided pediatric patients into age groups: 0-1, 2-5, 6-12, and 13-17 years [5,27]. Although pediatric patients aged 6 and 12 years were categorized in the same group in previous studies, our results suggested that those aged 6-7 and 10-11 years had different trends of several adverse events. Therefore, even more new findings might be obtained if previous studies were conducted by subdividing age categories. Previous studies have reported differences in the incidence of hospitalization and complication rates, treatment methods, and post-transplant follow-up among countries and regions in pediatric patients with renal transplants [26,27]. Therefore, findings from studies using databases of pediatric patients with renal transplants from a particular region may not apply to other regions.

The adverse events addressed in this study were previously known, and no new types of adverse events were reported. Thus, this study aligned with previous studies in terms of the types of adverse events reported. However, this study diverged from previous studies by investigating the association between these adverse events and age, uncovering several notable trends.

This study has several limitations. First, the incidence of adverse events could not be calculated because the FAERS database reported only the occurrence of adverse events. Second, the FAERS database may exhibit bias because it relies on spontaneous reports. This reporting mechanism may introduce various forms of bias, such as the characteristic that serious adverse events should be reported more comprehensively, while minor adverse events may be underreported relative to their actual occurrence. To illustrate, if one were to compare two adverse events where all occurrences are reported and another where only approximately half of the occurrences are reported discrepancy would introduce a substantial bias. This study focused on comparing the same types of adverse events across different age groups. Therefore, even if reporting bias exists between different types of adverse events, its effect on our inter-group comparisons should be relatively limited. Third, the data provided age at the occurrence of adverse events rather than age at renal transplant. Fourth, although the outcomes of pediatric patients after a renal transplant can vary depending on the donor’s age [28,29], the FAERS database did not provide information on donors. Fifth, when creating groups based on both age and country, the sample size for each group became too small. Therefore, this study prioritized setting groups based on age alone, as there are previous studies that have already conducted country-by-country analyses. In the univariate and multivariate linear regression analyses, we included countries with the first to third most reports as independent variables, but there were no significant differences between any of them. Although the small sample size in each country may have resulted in insufficient power, the results of this study may be applicable regardless of country or region, as the country effect is likely to be small compared to the effect of age. Sixth, FAERS data provides limited information. The cross-sectional nature of the FAERS data limits causal inferences. Therefore, there is a possibility of potential confounding factors, such as comorbidities. This study looked at trends in the occurrence of adverse events in two-year age increments. Even if there was a potential factor leading to higher or lower RP in only one age group, the trend would not be significant unless RP was higher in other age groups. Although the FAERS data does not allow for the examination of latent factors, this study may have been able to avoid the error of making a result statistically significant when it actually is not. Finally, because long-term immunosuppressant administration causes various adverse events, the relationship between the duration of immunosuppressant administration and adverse events should be investigated. However, because data on {start_dt}, {event_dt}, and {end_dt} were occasionally missing, the duration of immunosuppressant administration was unknown for several patients, thereby making the investigation of the relationship between the duration of immunosuppressant administration and adverse events impossible.

Contrarily, a strength of this study was the large sample that included numerous reports worldwide. The trends in the occurrence of each adverse event following immunosuppressant administration in pediatric patients with renal transplants could be investigated at two-year age increments. However, the evidence level may be low because the findings were based only on the FAERS data, which has several limitations, such as a lack of information on donors. Therefore, our findings need to be discussed in conjunction with the results of previous studies, such as those using another database, registry studies, and clinical trials. Furthermore, our findings should be confirmed through further studies.

## Conclusions

This study showed that there are various trends regarding the occurrence of each adverse event following immunosuppressant administration in pediatric patients with renal transplants. The observed trends could be categorized into several patterns. These include an increasing trend with age, a decreasing trend with age, a change in trend at a certain age, a scatterplot with a mountain-like shape, and a flat trend regardless of age. These diverse patterns highlighted that the types of adverse events requiring attention following immunosuppressant administration vary with age in pediatric patients with renal transplants. By incorporating our findings into clinical settings, healthcare providers can develop more nuanced and effective approaches to managing pediatric renal transplant patients. Our age-specific approach to adverse event monitoring and management has the potential to improve patient outcomes, reduce complications, and enhance the overall quality of life for pediatric patients with renal transplants.

## Appendices

Appendix A

Four patient background variables required data processing before being used in this study. The variable for {sex} was provided as {gndr_cod} for 2004Q1-2014Q2 and as {sex} for 2014Q3-2024Q1. We treated {gndr_cod} and {sex} as identical variables. Any {sex} data that was neither M nor F, or was missing, was classified as unknown. The unit of {age} was often in years, but for some patients, it was in decades, months, weeks, or days. When the unit of {age} was a decade, we multiplied the value by 10 and added 5 to estimate the {age}. When the unit of {age} was a month, we divided the value by 12 and rounded down to the nearest whole number. Similarly, when the unit of {age} was a week, we divided the value by (365.25/7) and rounded down to the nearest whole number. When the unit of {age} was a day, we divided the value by 365.25 and rounded down to the nearest whole number. In cases where the {age} was observational data and the unit of age was missing, we treated the unit of {age} as a year. The unit of {weight} was generally in kilograms, but for some patients, it was in pounds. When the unit of {weight} was in pounds, we converted it to kilograms by multiplying the value by 0.454. When the {reporter_country} was provided as COUNTRY NOT SPECIFIED, we classified it as unknown. 

Appendix B

**Table 3:** Trade and brand names of nine immunosuppressants.

Trade name	Brand name
(1) Prednisone, prednisolone, methylprednisolone	Aprednislon, cortancyl, decortin, medrol, prednisolon, prednison, prednisona, predonine, solupred, urbason
(2) Cyclosporine	Ciclosporin, ciclosporine, cyclosporin, equoral, gengraf, neoral, sandimmune
(3) Tacrolimus	Adoport, advagraf, astagraf, envarsus, graceptor, prograf, tacsant
(4) Mycophenolate mofetil	Cellcept, myfortic
(5) Everolimus	Afinitor, certican, rad001, sdz-rad, zortress
(6) Azathioprine	Azathioprin, azanin, azasan, imuran, imurek, imurel, zytrim
(7) Rituximab	Mabthera, rituxan
(8) Mizoribine	Bredinin
(9) Basiliximab	Simulect

Appendix C

The exclusion criterion is met when the following inequality is satisfied: {event_dt} < {start_dt}. However, we check if the inequality is satisfied or not as of January 1 of the year and as the first day of the month if only the year and only year and month are provided, respectively, for {start_dt}. We check if the inequality is satisfied or not as of December 31 of the year and as the last day of the month if only year and only year and month are provided, respectively, for {event_dt}.

## References

[1] Pattanaik D, Green J, Talwar M, Molnar M (2022). Relapse and outcome of lupus nephritis after renal transplantation in the modern immunosuppressive era. Cureus.

[2] Qayyum S, Shahid K (2023). Comparative safety and efficacy of immunosuppressive regimens post-kidney transplant: a systematic review. Cureus.

[3] Chandrashekhar P, Kaul A, Bhaduaria D (2022). Risk of tuberculosis among renal transplant recipients receiving rituximab therapy. Transpl Infect Dis.

[4] Dai R, Li J, Wu J (2021). Genetic and clinical determinants of mizoribine pharmacokinetics in renal transplant recipients. Eur J Clin Pharmacol.

[5] Moudgil A, Martz K, Stablein DM, Puliyanda DP (2011). Good outcome of kidney transplants in recipients of young donors: a NAPRTCS data analysis. Pediatr Transplant.

[6] Ernest TB, Elder DP, Martini LG, Roberts M, Ford JL (2007). Developing paediatric medicines: identifying the needs and recognizing the challenges. J Pharm Pharmacol.

[7] Ogura T, Shiraishi C (2024). Analysis of adverse events following phenobarbital administration for pediatric patients categorized by one-year age increments using the U.S. Food and Drug Administration Adverse Event Reporting System. Cureus.

[8] (2024). FDA: Adverse event reporting system. https://fis.fda.gov/extensions/FPD-QDE-FAERS/FPD-QDE-FAERS.html.

[9] Ogura T, Shiraishi C, Urawa A (2023). Analysis of death avoidance by concomitant use of prednisone in patients with renal transplant using the Food and Drug Administration Adverse Event Reporting System. Transpl Immunol.

[10] Ogura T, Shiraishi C (2024). Efficacy of prednisone avoidance in patients with liver transplant using the U.S. Food and Drug Administration Adverse Event Reporting System. Cureus.

[11] (2024). Drugs.com: Prednisone side effects. https://www.drugs.com/sfx/prednisone-side-effects.html.

[12] (2024). Drugs.com: Tacrolimus side effects. https://www.drugs.com/sfx/tacrolimus-side-effects.html.

[13] (2024). Drugs.com: Mycophenolate mofetil side effects. https://www.drugs.com/sfx/mycophenolate-mofetil-side-effects.html.

[14] Rodríguez Faba O, Boissier R, Budde K (2018). European Association of Urology guidelines on renal transplantation: update 2018. Eur Urol Focus.

[15] Sancho A, Gavela E, Avila A, Morales A, Fernández-Nájera JE, Crespo JF, Pallardo LM (2007). Risk factors and prognosis for proteinuria in renal transplant recipients. Transplant Proc.

[16] Höcker B, Fickenscher H, Delecluse HJ (2013). Epidemiology and morbidity of Epstein-Barr virus infection in pediatric renal transplant recipients: a multicenter, prospective study. Clin Infect Dis.

[17] Rysz J, Franczyk B, Radek M, Ciałkowska-Rysz A, Gluba-Brzózka A (2021). Diabetes and cardiovascular risk in renal transplant patients. Int J Mol Sci.

[18] Hsiao WC, Abt P, Amaral S, Levine M, LaRosa C (2022). Late renal allograft torsion in a pediatric transplant recipient. Pediatr Transplant.

[19] Ogura T, Shiraishi C (2023). Comparison of adverse events occurred during administration of dipeptidyl peptidase-4 inhibitor in patients with diabetes using FDA Adverse Event Reporting System. Clin Drug Investig.

[20] Sözen H, Fidan K, Özen O, Söylemezoğlu O, Dalgıç A (2019). Surgical complications after pediatric renal transplant. Exp Clin Transplant.

[21] Carraro A, De Gaspari P, Antoniello B (2024). New insights into pediatric kidney transplant rejection biomarkers: tissue, plasma and urine microRNAs compared to protocol biopsy histology. Int J Mol Sci.

[22] Lee RA, Gabardi S (2012). Current trends in immunosuppressive therapies for renal transplant recipients. Am J Health Syst Pharm.

[23] Spinner JA, Denfield SW (2022). Immunosuppressant drugs and their effects on children undergoing solid organ transplant. Pediatr Rev.

[24] Katz DT, Torres NS, Chatani B (2020). Care of pediatric solid organ transplant recipients: an overview for primary care providers. Pediatrics.

[25] Georgountzou A, Papadopoulos NG (2017). Postnatal innate immune development: from birth to adulthood. Front Immunol.

[26] Harambat J, van Stralen KJ, Schaefer F (2013). Disparities in policies, practices and rates of pediatric kidney transplantation in Europe. Am J Transplant.

[27] Kim JK, Lorenzo AJ, Tönshoff B (2022). Hospitalization following pediatric kidney transplantation: An international comparison among a Canadian pediatric transplant center, North American Pediatric Renal Trials and Collaborative Studies, and Cooperative European Pediatric Renal Transplant Initiative registry data. Pediatr Transplant.

[28] Opelz G, Döhler B (2010). Pediatric kidney transplantation: analysis of donor age, HLA match, and posttransplant non-Hodgkin lymphoma: a collaborative transplant study report. Transplantation.

[29] Chesnaye NC, van Stralen KJ, Bonthuis M (2017). The association of donor and recipient age with graft survival in paediatric renal transplant recipients in a European Society for Paediatric Nephrology/European Renal Association-European Dialysis and Transplantation Association Registry study. Nephrol Dial Transplant.

